# Feasibility and Characterization Mortar Blended with High-Amount Basic Oxygen Furnace Slag

**DOI:** 10.3390/ma12010006

**Published:** 2018-12-20

**Authors:** Wei-Ting Lin, Chia-Jung Tsai, Jie Chen, Weidong Liu

**Affiliations:** 1 Department of Civil Engineering, National Ilan University, No. 1, Sec. 1, Shennong Rd., I-Lan 260, Taiwan; jchen@niu.edu.tw; 2 ARDEX Taiwan Inc., 10F.-2, No. 120, Qiaohe Rd., Zhonghe Dist., New Taipei City 235, Taiwan; newbird4@gmail.com; 3 Department of Civil Engineering, University of Shanghai for Science and Technology, 516 Jun Gong Road, Shanghai 200093, China; wdliu2010@126.com

**Keywords:** basic oxygen furnace slag, supplementary cementitious materials, cementitious material, blended materials

## Abstract

Basic oxygen furnace slag (BOFS) was ground to three levels of fineness as a replacement for cement at weight proportions of 10, 30, 50, and 70 wt.%. Fineness and weight proportion were shown to have significant effects on the flowability and setting time of the mortars. The expansion of BOFS mortars increased with an increase in the proportion of cement replaced, thereby exacerbating the effects of cracking. Optimal mechanical properties were achieved when 10 wt.% of the cement was replaced using BOFS with fineness of 10,000 cm^2^/g. The compressive strength of BOFS mortar is similar to that of ordinary Portland mortar, which makes BOFS suitable for the partial replacement of cement as a supplementary cementitious material. Scanning electron microscopy results revealed that the reaction of CaO with H_2_O results in the formation of C–S–H colloids, whereas the reaction of SiO_2_ with Al_2_O_3_ produces C–A–S–H colloids. The use of BOFS as a partial replacement for Portland cement could make a tremendous contribution to the steel industry and help to lower CO_2_ emissions.

## 1. Introduction

The World Steel Association reported that global steel production increased from approximately 751 million tons in 1996 to more than 1630 million tons in 2017. The steel output of China (approximately 808 million tons in 2017) accounts for nearly half of global production [1]. Unfortunately, steel production has a negative impact on the environment.

General furnace slag refers to the by-products of extracting and smelting ore, and the various types of furnace slag refer to the refining method by which they were derived. Iron smelting involves the use of blast furnaces, which produces blast furnace slag (BFS). Steel manufacturing produces blast oxygen furnace slag (BOFS) and electric arc furnace slag (EAFS), which can be further divided into oxide ballast and reducing ballast, based on the smelting process [2,3,4]. Due to its hardness, BFS is commonly used for road construction and as concrete aggregate [5,6,7,8,9]; however, it must be stabilized first due to the presence of SiO_2_, CaO, Al_2_O_3_, and MgO. The reaction of free calcium oxide (f-CaO) with water can lead to the formation of Ca(OH)_2_ and CaCO_3_ [10,11], resulting in excessive expansion and alkali-aggregate reactions [12,13].

The explosion of the world’s population in the past few decades has created the problem of housing shortages [14,15,16], and the cement industry is one of the largest industrial sources of CO_2_ emissions [17,18]. As with Portland cement, furnace slag contains large quantities of SiO_2_ and CaO, [19,20,21,22] which means that when correctly ground, it can be used as a replacement for Portland cement. Most buildings are built using cement, the production of which creates massive amounts of CO_2_, the leading culprit in the greenhouse effect [23]. Cutting down on cement use would not only reduce CO_2_ emissions and slow down the greenhouse effect but also promote energy conservation. For this reason, developing supplementary cementitious materials and promoting the utilization of industry by-products can effectively benefit the conservation of limited natural resources and CO_2_ reduction [24].

In the past several years, the use of BOFS incorporated into cement-based mixtures as a partial replacement for cement or natural aggregates has been extensively investigated. Ding et al. indicated that BOFS can be used to replace natural aggregates due to its better mechanical properties and showed that no toxic materials exist in BOFS [25]. There have been many studies on the use of steel slag as aggregates in concrete, and its advantageous properties as an appropriate treatment for solving the expansion problem have been widely reported [26,27]. BOFS also can be treated as a cementitious material due to its silicate calcium minerals; however, previous studies indicated that the compressive strength and permeability of mortar or concrete containing BOFS tended to be negatively affected [28,29]. Although its application as a partial replacement for aggregates in conventional concrete, roller-compacted concrete, or self-compacting composites [30,31,32] has been widespread in the concrete industry, it is also suitable as a partial replacement for cement to solve the greater demand for cement for sustainable materials. Using finer BOFS particles in composites may be an effective strategy to compensate for the reduced strength of BOFS composites. The inclusion of high-amount BOFS (70% replacement of cement) in cement-based composites was also tested and evaluated.

In this study, we investigated the use of BOFS as a cement replacement in a blended mixture, with the fineness and proportion of BOFS used as variables in the experiments. Mortar specimens were subjected to a range of laboratory tests, including flow tests, setting time, compressive strength, and shrinkage. We also examined the formation of hydration products in mortar blended with BOFS.

## 2. Materials and Methods

### 2.1. Materials

**Figure 1:**
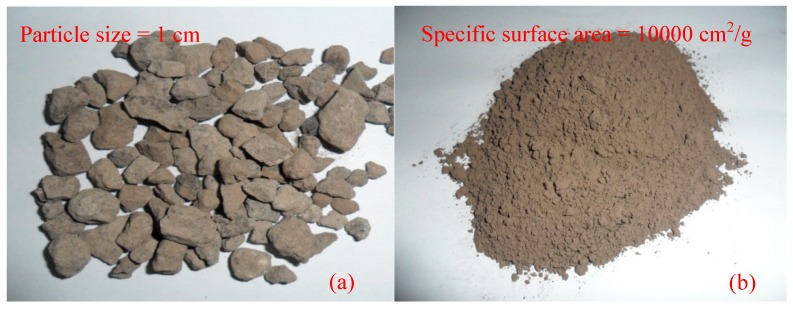
Appearance of BOFS before grinding (**a**) and after grinding (**b**).

**Figure 2:**
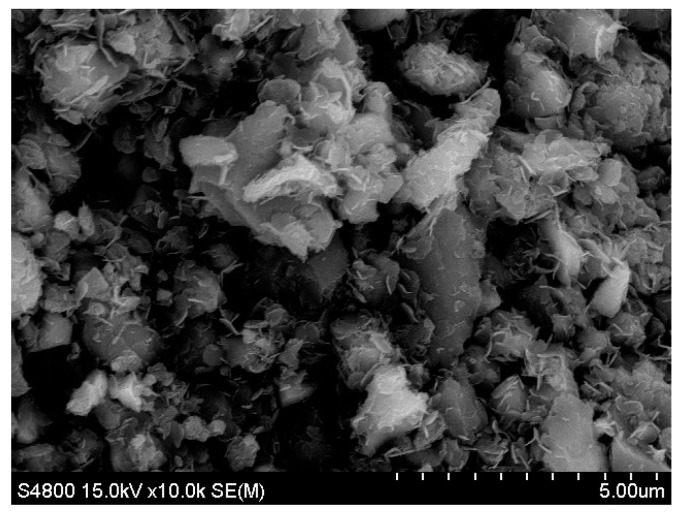
SEM image of BOFS after grinding.

**Figure 3:**
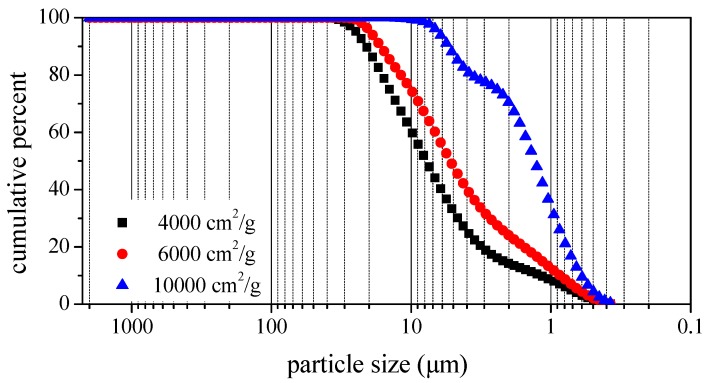
Particle size distributions of BOFS.

This study used Type-I Portland cement conforming to ASTM C150. The density of the cement was 3150 kg/m^3^ and the specific surface area of the cement was 3713 cm^2^/g. The chemical properties of the cement and BOFS are listed in Table 1. The loss on ignition of cement and BOFS was 1.38% and 0.83%, respectively. The BOFS is currently ground in ball mills. The BOFS used in this study had a density of 3590 kg/m^3^ and the specific surface area of BOFS was 4000, 6000, and 10,000 cm^2^/g using the nitrogen adsorption isotherms test (BET algorithm). The maximum grain sizes of BOFS passing through 45 μm, 35 μm, 15 μm were 4000 (average from 3500~4500), 6000 (average from 5500~6500), and 10,000 cm^2^/g (average from 7000~10,000) particles, respectively. The fine aggregates were used with a fineness modulus of 2.0, density of 2550 kg/m^3^, and absorption of 2.0%. The pozzolanic strength activity index of BOFS in accordance with ASTM C311 was 62% and 74% for testing ages of 7 and 28 days, respectively. As shown in Table 1, the chemical composition included CaO, Fe_2_O_3_ and SiO_2_. Figure 1 presents a photographic image of the BOFS and Figure 2 presents an SEM image showing angularity of the particles. The particle size distributions of BOFS are illustrated in Figure 3.

### 2.2. Mix Designs and Specimens

The water/binder mass ratio (w/b) of the mortar specimens was maintained at a constant 0.5, whereas the cementitious materials/fine aggregate mass ratio was 1:2.75 in accordance with the ASTM C109 specification. Table 2 lists the mix design of mixtures with 10%, 30%, 50%, and 70% BOFS (by weight) as a replacement for cement. OPM denotes ordinary Portland mortar; B refers to specimens containing BOFS; and 1, 3, 5, and 7 indicate specimens in which BOFS was used as a replacement for 10%, 30%, 50%, and 70% of the cement, respectively. In addition, three specimens were tested for each mixture in each test, and then the results were averaged and compared. A standard deviation was controlled less than 10% for the tested results.

### 2.3. Test Methods

Flow tests were conducted on BOFS specimens in accordance with ASTM C230, and setting times were tested in accordance with ASTM C191. The hydrated temperature was measured using semi-adiabatic calorimetry. One thermocouple was installed in the center of the insulating flask with the specimens. Each mixture of mortar consisted of 100 g blended materials and 50 g water and the paste was stored in the test room at a temperature of 23.0 °C and relative humidity of 80%. The hydrated temperature of the blended paste was recorded from 0 to 60 min using a data logger. Drying shrinkage tests were conducted in accordance with ASTM C490 using rectangular specimens (285 × 25 × 25 mm) in lime-saturated water for 72 h. Three specimens of each mixture were tested at 3, 7, 14, and 28 days. During curing, the temperature of the specimens was maintained at 23.0 °C ± 4 °C and the relative humidity was at least 80%. Compressive strength tests were conducted in accordance with ASTM C109 using cubic specimens (50 × 50 × 50 mm) at 7, 14, and 28 days. Three specimens of each mixture were tested. Scanning electron microscopy (SEM) analysis was conducted on representative samples of 1 mm × 1 mm × 1 mm, in accordance with ASTM C1723. Table 3 presents the tests performed, the dimensions of the specimens and the standards.

## 3. Results and Discussion

### 3.1. Effect of BOFS on Setting Time

**Figure 4:**
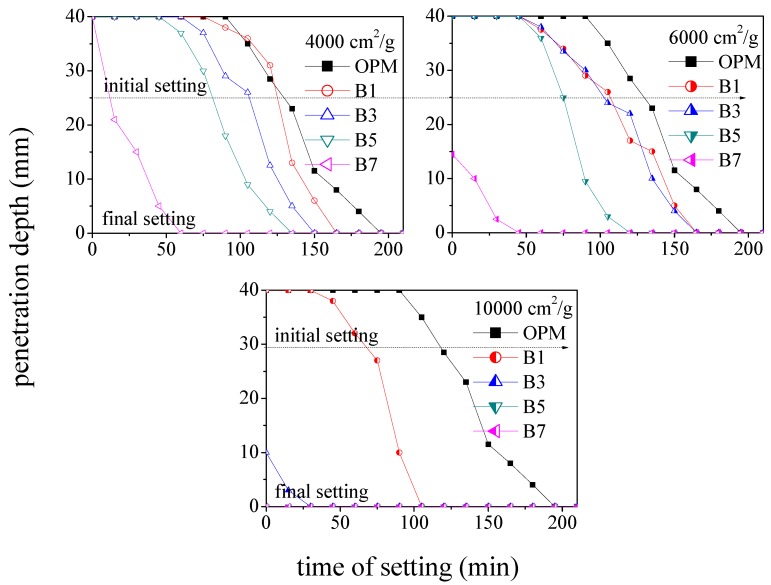
Setting times of samples made with BOFS of various specific surface areas.

Figure 4 illustrates the setting time vs. penetration depth in samples prepared using BOFS of various specific surface areas. An increase in the specific surface area was shown to decrease the initial and final setting times. Figure 4 also illustrates the influence of BOFS content (surface area of 4000 cm^2^/g) on setting times. An increase in BOFS content was shown to reduce the initial setting time. Sample B7 set in just 45 min, while OPM specimens required 195 min for 6000 cm^2^/g. Compared to OPM specimens, the setting times were reduced as follows: 50% BOFS (15%), 30% BOFS (23%), and 10% BOFS (30%). In conclusion, the setting time of B5 and B7 specimens approached less than 1 min when the penetration depth was close to 0 mm. Thus, the B5 and B7 specimens presented a rapid hardening condition.

**Figure 5:**
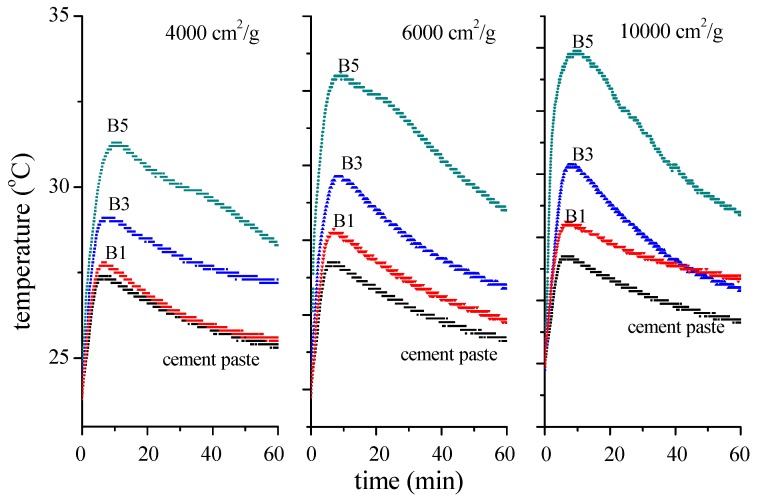
Hydrated temperature of BOFS samples of various specific surface areas.

Figure 5 illustrates the temperature of the specimens measured during the hydration process for 1 h. It indicates that the BOFS replacement temperature increased as the hydrated temperature increased, as well as with increases in the specific surface area. As illustrated, the highest temperature was at the hydrated time of 600 s, which was set as the period of initial hydrolysis; however, the temperature suddenly dropped between 600 and 6000 s hydrated time. The hydrated temperature of the cement pastes was 28 °C and BOFS pastes performed at higher temperatures. In particular, for the B5 specimens with a specific surface area of 10,000 cm^2^/g, the hydrated temperature was raised to 34 °C. It also presented a rapid hardening condition, which was consistent with the setting time due to the higher hydrated temperature. According to the literature [33], higher temperatures enable BOFS pastes to show rapid hydrated reactivity, which indicates that the calcium-silicate-hydrate (C–S–H) identified in BOFS pastes may be produced by the reaction between free lime and quartz. Higher hydrated temperatures were observed during the blending process for B5 and B7 specimens with a rapid hardening condition. Larger proportions of BOFS decreased the setting time. The CaO in BOFS reacted violently with water to generate Ca(OH)_2_ and a larger specific surface area led to a more violent reaction [34]. Therefore, as inferred from the aforementioned properties, BOFS accelerated the setting of blended cements.

In addition, the specific surface area of BOFS particles increased as the reaction area with cement as blended materials increased. The flowability of blended materials is dependent on the particle size and it has also been observed that the degree of hydration of paste and mortar is improved by better particle size distribution and finer particle sizes of cementitious material [35]. Due to the finer particle sizes of BOFS as illustrated in Figure 3, the fineness of BOFS increased as the reaction speed of the blended materials increased, resulting in rapid hardening. More than 70% replacement with BOFS caused the phenomenon of flash setting in which C_3_A reacted with water causing the liberation of a high amount of heat, and rapid setting between cement and BOFS. On the basis of the X-ray diffraction analysis from a previous study [36], more C_3_A reacted from BOFS and enabled the rapid hardening and higher hydrated temperature. 

**Figure 6:**
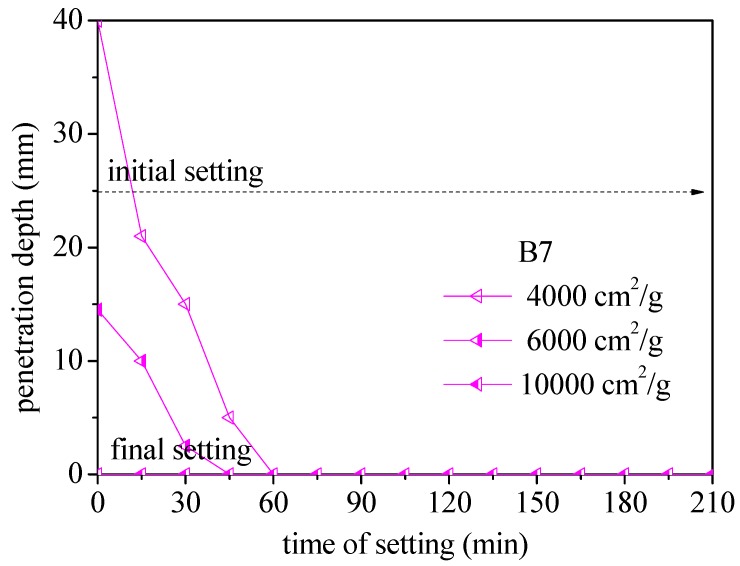
Setting times of B7 specimens using BOFS of various specific surface areas.

Figure 6 illustrates the setting times of samples in which 70% of the cement was replaced with BOFS (B7) with specific surface areas. The initial and final setting times of BOFS specimens with a specific surface area of 10,000 cm^2^/g was on the scale of several seconds, which is far lower than those of OPM specimens. The other setting times using 70% cement replacement were as follows: 6000 cm^2^/g (45 min) and 4000 cm^2^/g (60 min). These results indicated that a larger specific surface area accelerated the setting time, especially for B5 and B7 specimens. These are available as rapid hardening materials containing fibers for emergency repair.

### 3.2. Effects of BOFS on Flowability

**Figure 7:**
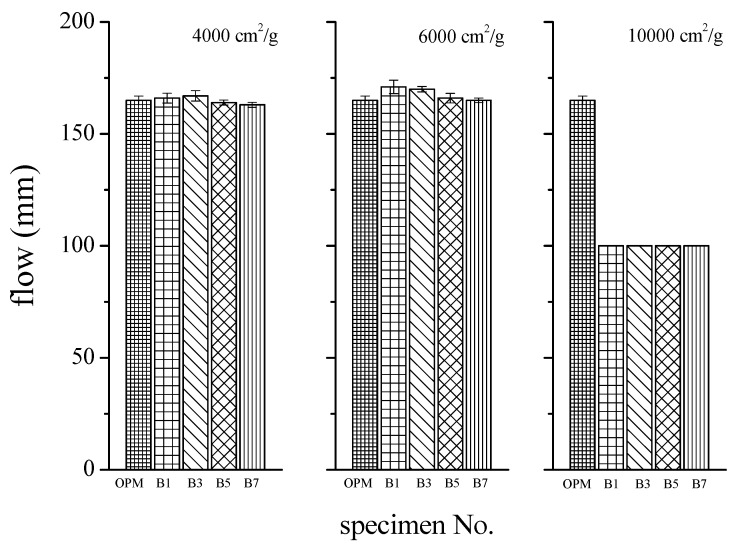
Flow test results of BOFS specimens.

**Figure 8:**
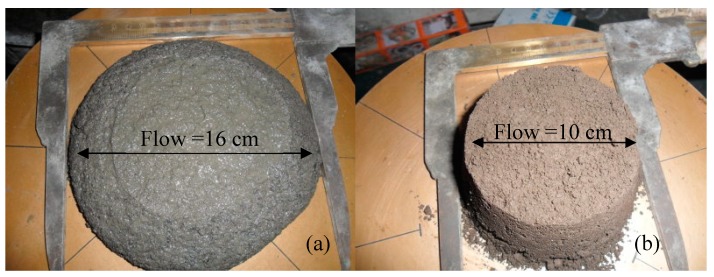
Mortar specimens used in flow tests: (**a**) ordinary Portland mortar, OPM; (**b**) specimen with 70% of 10,000 cm^2^/g BOFS.

A constant water-to-binder ratio of 0.5 was maintained in the preparation of mortar specimens for flow tests referring to the ASTM C109 standard. The results are presented in Figure 7, with the X-axis indicating differences in the specific surface area of BOFS and the Y-axis indicating flowability (cm). An increase in the specific surface area of BOFS was shown to decrease flowability. Only slight differences in flowability were observed when the quantity of 4000 and 6000 cm^2^/g BOFS (as a replacement for cement) was varied; i.e., the flowability of samples B1, B3, B5, and B7 was approximately the same as that of the control specimens with 160 ± 5 mm due to the 0.5 of w/b (higher than the standard value with 110 mm). When the specific surface area of BOFS exceeded 10,000 cm^2^/g, the flowability decreased significantly. The liquidity of the B7 specimens remained almost unchanged, due to the rapid hardening, as shown in Figure 8. The rapid shaping of B7 specimens resulted in maintaining flowability. This meant that the amount of BOFS replacement was not a significant factor for flowability, whereas fineness was a significant factor for flowability. This is due to the fact that the adsorption of mixing water was higher in the finer BOFS grains. This also occurred because of the increasing water requirement of the mixture caused by the higher surface area of the finer BOFS particles. According to a previous study [37], the flowability of cement-based composites increased along with the fineness of the cementitious material. Another study [38] stated that the finer cementitious material hydrated more quickly, and the hydration temperature increased more rapidly, which is consistent with the results in this study.

Overall, these results show that the quantity of BOFS in mortar specimens has a negligible effect on flowability; however, the specific surface area has a significant effect due to the larger surface fineness and higher packing density of BOFS particles. The results are comparable with a similar study of cement pastes, which found that a higher specific surface area of blended particles can result in a reduction in the paste film thickness surrounding the solid particles, thus leading to lower workability or higher water/admixture demand [39]. It also indicated that the small and angular particles of the BOFS can reduce the friction between particles and fill the voids between solid particles, thus leading to a decrease in the minimum water needed to initiate flow. The influence on flowability was probably due to the amount of the cement replacement, the rough surface and the fineness of the BOFS particles [40].

### 3.3. Effects of BOFS on Length Change

**Figure 9:**
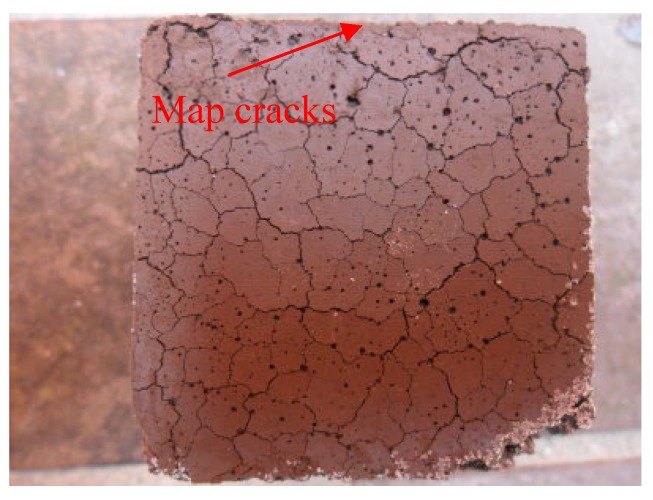
Image of B5 specimen showing obvious cracking.

**Figure 10:**
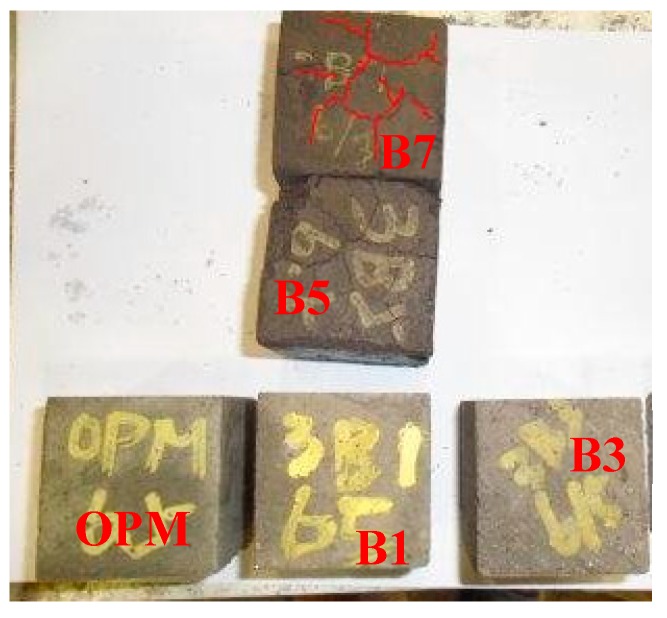
Image of OPM, B1, B3, B5 and B7 specimens.

**Figure 11:**
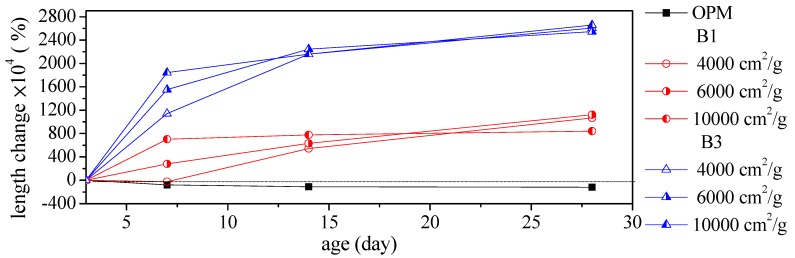
Shrinkage trends in samples with various quantities of BOFS and various specific surface areas.

Figure 9 presents an image of a 50% BOFS specimen which demonstrated obvious cracking. We therefore focused on specimens in which a smaller percentage of the cement was replaced with BOFS (10% and 30%) and the images of OPM, B1, B3 and B7 are shown in Figure 10. These indicated that the 70% BOFS specimen also presented obvious cracks due to the quickly hydrated reaction and high levels of volume expansion. This result is in accord with those obtained in the previous research [41,42] and indicates that the free CaO and MgO contents of the BOFS have an important role in causing the instability of length change [41,42]. Figure 11 presents the length change results of the BOFS specimens, indicating that a higher BOFS content led to higher shrinkage after drying. The degree of shrinkage was largely independent of the specific surface area.

Figure 11 illustrates the shrinkage trends in specimens prepared using BOFS with various specific surface areas in various quantities. The black solid line in Figure 11 indicates OPM specimens; the red solid line indicates B1 specimens with BOFS of various specific surface areas; and, the blue solid line indicates B3 specimens with BOFS of various specific surface areas. Positive values indicate expansion and negative values indicate shrinkage. Overall, as Figure 11 shows, it was found that only OPM specimens demonstrated substantial shrinkage. Conversely, all the mortar specimens including BOFS consistently demonstrated the expansion phenomenon, but specific surface areas had no apparent effect on expansion. At 28 days, the expansion ratio of the B3 specimens exceeded that of the B1 specimens by two times.

### 3.4. Compressive Strength

**Figure 12:**
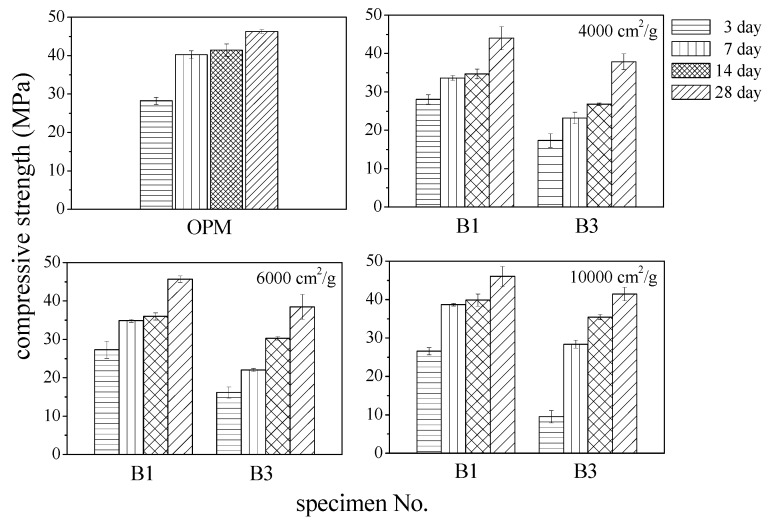
Compressive strength of BOFS blended mortar.

Figure 12 presents the compressive strength of specimens made with 10% and 30% BOFS with specific surface areas. The results indicate a relationship between compressive strength and cement replacement. Compressive strength was shown to increase with age, regardless of the fineness of BOFS specimens, presenting a similar trend to the OPM specimens. Replacing 10% of the cement with BOFS resulted in mortar with the highest compressive strength, regardless of fineness. Specimens made with 10,000 cm^2^/g BOFS attained the strength of OPM at 28 days. The strength of specimens made with 6000 cm^2^/g and 4000 cm^2^/g BOFS was only 2% and 5% lower than that of OPM, respectively. Figure 12 illustrates the relationship between compressive strength and cement replacement using 4000 cm^2^/g BOFS. The results indicated that a curing age of 7 to 28 days showed the same trend and strength significantly declined when BOFS exceeded 10 wt.%. At 28 days, the strength of B1 was approximately 5% lower than that of OPM, which demonstrates that BOFS produced an effective hydration reaction and packing (compaction) effect. In conclusion, the composites containing 10% BOFS demonstrated better performance on compressive strength, attaining 95% of the strength of OPM specimens. Previous studies also indicated the same trends; that is, the compressive strength was shown to decrease as the substitution ratio of BOFS increased, and the optimum percentage of BOFS was about 10% of the binder materials [29,43,44].

### 3.5. SEM Observations

**Figure 13:**
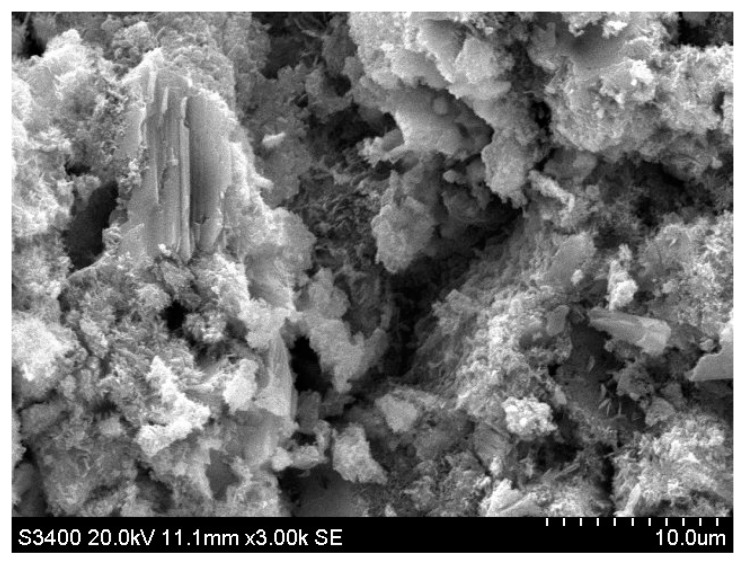
SEM image of OPM specimen.

**Figure 14:**
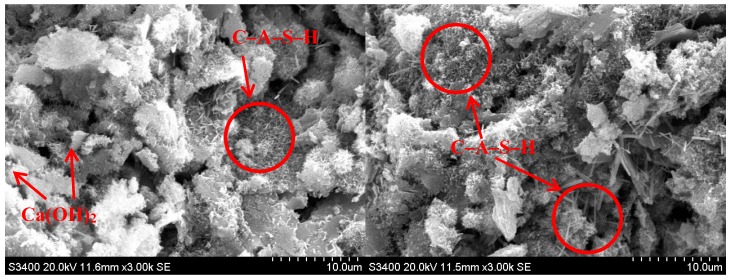
SEM images of B3 specimens that included BOFS with a fineness of 10,000 cm^2^/g.

The phase structure was altered by mixing BOFS with cement through a hydration reaction, as shown in the enlarged images of OPM (Figure 13) and B3 (Figure 14). The main hydration products on the surfaces of B3 specimens were calcium-silicate-hydrate (C–S–H) and calcium–aluminum–silicate–hydrate (C–A–S–H) as well as Ca(OH)_2_ that appeared in the microstructure. This may have been due to an insufficient quantity of BOFS reacting with Ca(OH)_2_. The pores in the B3 samples were filled primarily with C–A–S–H or C–S–H colloids. We observed a number of polygonal column-like (ettringite) structures, composed of Ca, S, Al, and O. We also observed ettringite structures on the surface of B3 specimens, as shown in Figure 14. This was a C–S–H type phase containing Al, Mg and Fe, which is consistent with previous studies [33,36,45,46,47,48].

Based on the SEM micrographs, we deduced that the reaction mechanism was as follows: CaO in cement reacted violently with water to generate Ca(OH)_2_. Consequently, increasing proportions of BOFS accelerated the hardenability of cement paste which is consistent with the results of the test for setting time. In the next phase, Ca(OH)_2_ reacted with SiO_2_ or Al_2_O_3_ to form C–S–H or C–A–S–H colloids, which may be a second hydrated reaction. The iron oxide content was approximately 32% and the manganese oxide content was approximately 6–7%, as shown in Table 1. It was therefore surmised that the cementitious properties of BOFS can be attributed primarily to C–A–S–H and C–S–H.

## 4. Conclusions

1. Fineness of the BOFS was the primary factor influencing the fluidity of BOFS mortar. Inclusion of 10,000 cm^2^/g BOFS had a particularly pronounced effect in reducing fluidity. Finer BOFS can reduce the friction between particles and fill the voids between solid particles, thus leading to a decrease in the minimum amount of water needed to initiate flow.
2. Increasing the amount of BOFS used as a replacement for cement was shown to speed up the initial and final setting times. The B7 specimens with 6000 cm^2^/g completed final setting within 45 min. This is 69% faster than OPM specimens (195 min); B5 was 30% faster, B3 was 23% faster, and B1 was 15% faster.
3. Replacement of cement with BOFS resulted in increased expansion and cracking, particularly when BOFS was used to replace more than 50%. At 28 days, the expansion ratio of the B3 specimens exceeded that of the B1 specimens by two times.
4. BOFS with a specific surface area of 10,000 cm^2^/g attained compressive strength values close to those of OPM specimens. BOFS with a specific surface area of 6000 cm^2^/g produced mortar that was 2% weaker than OPM, whereas BOFS with a specific surface area of 4000 cm^2^/g produced mortar that was 5% weaker.
5. Compression strength of the mortar specimens decreased with an increase in cement replacement. Specimens that included 10% BOFS achieved the highest compressive strength at 28 days; however, even this was 5% lower than that of OPM. Increasing BOFS content beyond 50% greatly decreased compressive strength due to increased expansion and cracking. However, the inclusion of fiber in 10% BOFS composites may be used as repair mortar in emergency engineering, especially for BOFS with 6000 cm^2^/g due to their rapid hardening.
6. SEM analysis revealed that the CaO component of cement reacts with H_2_O, SiO_2_, and Al_2_O_3_ from BOFS to mainly react C–S–H and C–A–S–H, which was a major source of strength development in the BOFS blended materials.

## Figures and Tables

**Table 1:** Chemical composition of cement and basic oxygen furnace slag (BOFS).

Chemical Composition (wt.%)	Cement	BOFS
SiO_2_	21.04	12.20
Al_2_O_3_	5.46	4.76
Fe_2_O_3_	2.98	32.20
CaO	63.56	38.90
MgO	2.52	6.26
MnO	-	2.39

**Table 2:** Mix designs (kg/m^3^).

Min No.	w/b	Water	Cement	BOFS	Fine Aggregates
OPM	0.5	258	516	-	1421
B1		259	466	52	1422
B3		260	364	165	1428
B5		261	261	261	1435
B7		261	157	367	1441

**Table 3:** Test methods.

Test Target		Specimen Dimensions (mm)	Referenced Standard
Fresh properties	Flow test	-	ASTM C230
	Setting test	-	ASTM C191
	Hydrated temperature test	-	-
Mechanical properties	Compressive strength test	50 × 50 × 50	ASTM C109
Durability	Drying shrinkage test	285 × 25 × 25	ASTM C596
Micro-structure observations	SEM observation	10 × 10 × 3	ASTM C1723
